# Correction: Low-energy electron distributions from the photoionization of liquid water: a sensitive test of electron mean free paths

**DOI:** 10.1039/d5sc90072g

**Published:** 2025-03-25

**Authors:** Titouan Gadeyne, Pengju Zhang, Axel Schild, Hans Jakob Wörner

**Affiliations:** a Laboratory for Physical Chemistry, ETH Zürich Vladimir-Prelog-Weg 2 8093 Zürich Switzerland pengju.zhang@phys.chem.ethz.ch hwoerner@ethz.ch; b Département de Chimie, École Normale Supérieure, PSL University 75005 Paris France

## Abstract

Correction for ‘Low-energy electron distributions from the photoionization of liquid water: a sensitive test of electron mean free paths’ by Titouan Gadeyne *et al.*, *Chem. Sci.*, 2022, **13**, 1675–1692, https://doi.org/10.1039/D1SC06741A.

The authors regret that an incorrect version of Fig. 4 was included in the original article. In the original article, the horizontal scale along the bottom plot of Fig. 4 spans a range from 0 to 50 eV, when it should read 0 to 1 eV.

The authors note that this error is only of graphical nature and does not affect the results and conclusions of the paper.

The correct version of Fig. 4 is presented here.

**Fig. 4:**
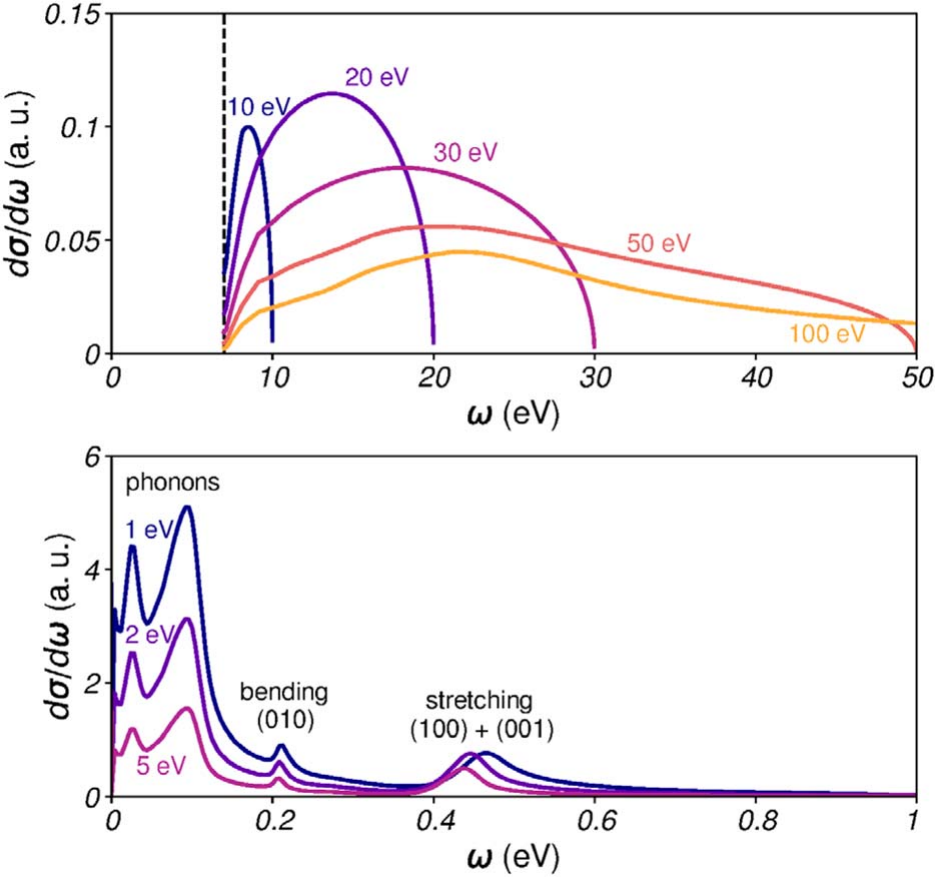
Singly-differential cross sections for inelastic scattering. (Top) SDCS for electronically inelastic events, for incident kinetic energies *E* = 10, 20, 30, 50 and 100 eV. The black vertical line marks the excitation threshold for liquid water. (Bottom) SDCS for vibrationally inelastic events, for *E* = 1, 2 and 5 eV.

The Royal Society of Chemistry apologises for these errors and any consequent inconvenience to authors and readers.

